# “Preventing the Pain” When Working with Family and Sexual Violence in Primary Care

**DOI:** 10.1155/2013/198578

**Published:** 2013-02-26

**Authors:** Jan Coles, Elizabeth Dartnall, Jill Astbury

**Affiliations:** ^1^Department of General Practice, Monash University, Building 1, 270 Ferntree Gully Road, Notting Hill, VIC 3168, Australia; ^2^Sexual Violence Research Initiative, Gender and Health Research Unit, Medical Research Council, Private Bag x385, Pretoria 0001, South Africa; ^3^School of Psychology and Psychiatry, Faculty of Medicine, Nursing and Health Sciences, Monash University, Clayton, VIC 3800, Australia

## Abstract

Primary care professionals (PCPs) are increasingly being expected to identify and respond to family and sexual violence as the chronic nature and severity of the long-term health impacts are increasingly recognized. This discussion paper reports the authors' expert opinion from their experiences running international workshops to prevent trauma among those who work and research sexual violence. It describes the burnout and secondary traumatic stress literature which provides the evidence supporting their work. Implications for practicing basic training in response to trauma and ongoing education are a key area for responding to family violence and preventing professional stress. A professional culture that supports and values caring well for those who have experienced family violence as well as “caring for the carer” is needed. Working in teams and having more support systems in place are likely to protect PCPs from secondary traumatic stress and burnout. Undergraduate and postgraduate training of PCPs to develop trauma knowledge and the skills to ask about and respond to family violence safely are essential. In addition, the healthcare system, workplace, and the individual practitioner support structures need to be in place to enable PCPs to provide safe and effective long-term care and access to other appropriate services for those who have experienced family violence.

## 1. Introduction

Being a primary care professional (PCP) means being at the forefront of managing chronic, complex, and difficult areas of health. Working with patients who have cancer, those who are dying and those who experience or have experienced family and/or sexual violence can be difficult and emotionally confronting. In a day's work, PCPs see many complicated and complex patients, but those who experience family violence can be particularly demanding and challenging because of chronic health issues, psychological distress, relationship and authority issues, and coping strategies such as denial or somatization [1]. If we then add to the potential emotional impact of survivor stories on PCPs, the mental and emotional work of caring for these patients, the patient-professional interaction and boundary issues, and the long-term nature of the care required [1], we will have the perfect recipe for increased PCP stress.

Work-related stress is already experienced by family physicians and nurses, with burnout being the most investigated area [4–7]. Prevalence rates of burnout for medical residents (including family medicine residents) have been reported between 18 and 82% [7] and between 10 and 55% for family physicians [4, 6, 7]. A number of factors have been identified as important contributors to burnout; these include the work environment, changing policy, changes in the practice of family medicine, workload, loss of autonomy, and feeling a lack of personal accomplishment [4, 7]. Personal characteristics of the PCPs themselves, such as being high achievers and perfectionists, the relationship between work and self-esteem, gender, age, and family and support networks, may also contribute to their susceptibility to stress [4, 7, 8].

PCP's recognition and support of patients who have experienced violence may improve the mental and physical health through early intervention and better service access [9–12]. 

Working with family violence can, in itself, be difficult and confronting for PCPs because of the patient's experience within their family. PCPs may also be both victims and perpetrators of family violence and this can influence their ability to work effectively with patients. A Canadian study of PCPs found that nearly 50% had experienced family violence themselves [13]. An earlier study by Sugg and Inui reported that 15% of male doctors and 31% of female doctors had a personal history of child abuse or intimate partner violence [14]. PCPs may be concerned that family violence is a private “no-go” area that cannot be discussed without offending the patient. They describe feeling overwhelmed and concerned about broaching the topic with patients, that they lack the time and the confidence to deal with the problem, that they might make the situation worse, and that they “cannot fix it” anyway [13, 15–17]. PCPs can find dealing with both the victim and the perpetrator difficult [15].

Personal safety issues often confront those who work with victimized children and adults. The traumatic nature of child abuse and adult violence with its negative effects on the emotional, mental, and physical health of victim/survivors is well documented. Less discussed is the impact of working in this area on the well-being of the medical professionals and their staff [18].

This paper outlines a theoretical framework for understanding the possible reactions of PCPs who work in this area. We then discuss the emotional and physical safety issues while being at work and the social, emotional, and physical impacts of child abuse and violence at work. It concludes with a discussion of strategies to reduce and manage potential trauma.

## 2. Understanding the Potential for Trauma

Researching and working with victims of family violence can be emotionally challenging [19–23]. Working with children can make it even more so because of bearing witness to the child's abuse experiences, conflicts with parents and carers, the inability to form an effective therapeutic relationship with the child or their family, a hostile working environment, lack of or failure of services to assist the child and the family, direct threats and concerns about personal safety, and the possibility of litigation [22, 24–26]. In addition, working with victimised children and adults can evoke strong emotional reactions [22, 26]. The general practitioners (GPs) below describe their encounters:

*A mother was breastfeeding when her son's smile triggered her memories of incest. She told me the story of her pain caused long ago by the CSA perpetrated by her father. Her childhood abuse was replayed across a generation. Her story was powerful, making me pay attention to her story amongst so many illness stories. As I reflect on that consultation now, I think it was the intensity of her fear and distress which alerted me to the importance of her experiences. She put her baby on one side of the consulting room, as far away as possible, and just cried. I did not know how to help her. (Female GP, urban) [27].*

*There were two boys and a little girl … and they all lived in the same street. I went and saw the parents of the two disabled boys [who were abusing the little girl] and they weren't prepared to do anything, they did not want to know about it, did not feel like supervising, perhaps they did not believe the story. I reported the case to child protection and the families all said “We have to move out of town if you cannot do anything and we do not want to leave. The kids do not want to leave the school and why should we have to go away to keep our children safe.” […] That process was extremely painful, you need to go through with the families, families of the abused and the family of the abuser and see it brought out in the open, to the police and then watch them go through the pain, through the system. (Female GP small rural town) [28].*

*Before I started doing work [with men who perpetrate violence], I can remember saying how lucky I was that I worked in an area where there was no abuse … what a clever decision I'd made, to pick such a good postcode, but obviously I just did not see it […] I think it's more that you are aware that it [abuse] goes on and I'm looking out for it more. In the past, if someone would say something, start hinting it, you'd run a million miles, you'd change the topic, you'd start talking about something else and it's easy to do that […](Male GP, urban) [28].*

There is an increasing literature on the emotional work of “caring”[29] and the toll this work takes on those who undertake it [18, 24, 30–32]. The effects of being a direct or indirect witness to child abuse and adult violence can result in compassion fatigue, burnout, vicarious trauma and secondary traumatic stress. These terms are often used interchangeably but are, in fact, conceptually different. 

Compassion fatigue is a general term applied to anyone who suffers as a result of acting in a helping capacity, a natural outcome of helping and knowing about trauma [33, page 92] and [34, page 9].

Burnout occurs when a person's health or outlook on life is negatively affected because of the impact of their work. Figley [34] described burnout as physical, emotional, and mental exhaustion caused by long-term involvement in emotionally demanding situations, as a gradual wearing down over time. Workers with burnout report low job satisfaction, feelings of powerlessness, and being overwhelmed at work [34]. 

Vicarious trauma is defined as “the transformation of the therapist's or helper's inner experience as a result of empathetic engagement with survivor clients and their trauma material” [30, page 31] and results in a profound shift in the worldview of the worker. The result of vicarious trauma can potentially include the disruption of the worker's view of themselves, “the self-changing nature of care giving” [31, page xxxviii], others and the world in general [35]. Child protection workers, physicians, nurses, therapists, social workers and child abuse researchers report such experiences. The traumatic experience can extend beyond those who work directly with the victim/survivor. Traumatization has been reported in those who have indirect contact: in receptionists, supervisors, and managers, in those processing accounts or records, in family members of the treating professionals, and in those working with patient histories or reports.

Vicarious trauma influences different people in different ways. The impact is related to the trauma they are exposed to, their own characteristics and history, the research methods they use, their support systems, and context in which they do their research [36]. It is described as a pervasive feature of working with the traumatised and a cumulative response to traumatic material. It can be triggered by either a one-off exposure to a significant issue or repeated exposure to a range of issues and incidents. It can have a profound impact on individuals and be as debilitating as the primary trauma [30]. Other contributing factors originally proposed by Pearlman and Saakvitne that apply to primary care across a range of settings internationally include the chronicity of the trauma work, combining service provision and research (particularly in resource poor nations), increased violence to women and children in war and conflict areas, the individual professional's capacity for empathy, and the individual's personal history of trauma [30]. The vicarious trauma has been criticised because it fails to explain why similar experiences cause vicarious trauma in some while others do not experience vicarious trauma.

Secondary traumatic stress is a similar concept to vicarious trauma, but there are differences as it is based on a set of clinical symptoms. Secondary traumatic stress closely parallels posttraumatic stress disorder in its symptomatology [37] and is a normal response to exposure to trauma through working with survivors of traumatic events [33]. Symptoms of secondary traumatic stress impact on psychological functioning, result from cognitive shifts and disturbances in relationships [2]. They include intrusive symptoms such as PCPs re-experiencing the survivors' trauma through thoughts, feelings, and images; avoidance and numbing symptoms such as avoiding working in areas that recall the trauma or forgetting parts of an interview; and symptoms of hyperarousal such as palpitations and sweating and nightmares and sleep disturbances [38, 39]. 

Vicarious trauma and secondary stress have provided the conceptual framework for much of the literature that underpins the current understanding of the impact on those who work with survivors of trauma. More recent work has highlighted methodological issues and the need to measure all of these areas [39] to test their independence. 

In medicine and nursing, the trauma literature is based on burnout as a response to caring. Burnout is defined as feelings of emotional exhaustion, depersonalization, and a perceived lack of personal accomplishment because of a long-term engagement with emotionally demanding situations [2, 4]. Burnout as a model has the advantage of combining the personal impacts and the impact on work, but the interrelationships between the three factors are unclear [2].

Preventing, understanding, and recognising vicarious trauma, secondary traumatic stress, and burnout in their work with family violence will assist PCPs to respond more effectively to it and to reduce its potential to adversely affect the way in which they work and their view of the world in general. For those already working in the area, it will assist them to better understand their experience and for those in management positions, to put in place strategies to prevent the possibility of secondary traumatization for themselves and their staff.

## 3. How Can PCPs Prepare to Work with Patients Who Have Experienced Family Violence?

PCPs must be trained to do this work at undergraduate and postgraduate levels and as part of their ongoing professional development because PCPs still report being inadequately trained to do so. Both training and professional experience have been associated with increased feelings of confidence in dealing with violence as well as increased comfort in discussions with patients making PCPs feel more in control and reducing PCP stress [5, 13, 40]. 

The institutions and professional organisations responsible for the education PCPs must ensure that students and professionals are trained well and develop the necessary knowledge skills and attitudes as well as the facilitating support for those working with family violence. Organisations which were expected to champion the health needs of women and children but failed to do so, by marginalising the need to respond to violence, blaming the victim, not addressing the training needs of PCPs, and discrediting the work of those responding to and supporting women were identified as particularly difficult for workers and researchers [41]. Hierarchical organisations have been identified as a more important predictor of trauma than the individual characteristics of the workers [42].

The education of PCPs should address the needs of patients first and foremost, but the PCPs sense of vulnerability, fear, and inadequacy also need to be addressed for them to work effectively in this area. Like other social problems and health risk behaviours, there is no easy “cure” to family violence and PCPs need to reframe what they consider as a “successful” consultation [43] rather than being frustrated by a lack of change [17]. PCPs report of positive changes after family violence is disclosed, including patient engagement with counselling and legal services [16].

*I asked a 40 year old new patient about child abuse which I was certain she had experienced. She denied ever experiencing abuse. I was left wondering what I could have done better. Twelve months later she came in and said “You know that question you asked me twelve months ago, I couldn't talk then but I can today.” In retrospect my first consultation was very successful, I just did not know it. (Female GP, urban).*

Traumatic reactions are related to the duration of exposure to traumatic material and the type of trauma experienced [5]. While PCPs cannot control the content on acute consultations, they can manage and structure their work in terms of review appointments and should consider structuring their work so that they have regular breaks and opportunities to manage less demanding problems. Support and supervision from more experienced or staff trained in responding to trauma may help in the management of distressing cases.

PCPs need to reflect on their ability to provide care for those who have experienced family violence. While all should have a basic competency in recognising and responding to violence in positive and helpful ways, not all may be willing or able to deliver care. In such cases, knowledge of appropriate providers of ongoing care and support services is essential. 

Support systems and networks for PCPs need to be considered because these are often not routinely available to PCPs. Setting up a recognised “family violence team” at work for all who work with family violence is useful, providing peer-peer support and the ability to discuss difficult or distressing cases [1]. Psychiatric and counselling services have a much more developed culture of providing support and supervision for those working with mental illness and traumatized clients. PCPs may need to consider engaging with services outside of work for support and supervision if they are going to regularly work with family violence. Networks can include coworkers within primary care and may extend to other agencies working with those who experience violence; working as part of a “team” is often a much more positive experience than working alone. Support systems that extend beyond the clinic should not be forgotten, with friends and family being reported as an important support [5, 41].

## 4. Preventing Burnout and Vicarious Trauma among PCPs

*“A key factor in the ability to care for others is the ability to care for oneself” page 106 [44].*

Working with family and sexual violence can be difficult. Although not all of us working with trauma will experience secondary trauma, we are all at risk [45]. Acknowledging how emotionally difficult working in this field can be, recognising that it will affect and may distress you, learning how to recognise symptoms of secondary trauma early and the importance of self-care are essential elements in managing and preventing secondary trauma. 

The literature highlights a number of responses that can be used at educational, organizational, and personal levels to prevent and respond to secondary trauma, and in turn, improve the quality of care provided by PCPs to people experiencing family and sexual violence.

*At an educational level*

- An understanding of burnout, secondary, or vicarious trauma and their implications should be integrated into training curricula of all helping professionals working with family and sexual violence [44, 46–48].

*At an organisational level*

- Develop a workplace policy that recognises secondary trauma as a potential workplace health risk; addresses related stigma; normalises work place stress; and outlines appropriate responses, including professional development; workload and case management; peer support and ongoing routine management meetings in which secondary trauma is discussed [48–50].
- Develop a culture of learning, sharing and support within the workplace [44, 48].
- Provide adequate support and supervision which includes discussion of secondary trauma and its implications [46].
- Provide workplace development programmes that include secondary trauma and how to identify early warning signs between oneself and others [46, 49].
- Ensure managers who are appropriately trained to recognise and respond to secondary trauma in a supportive, nonpunitive manner [49].
- Review workplace policies regularly.

*At an individual level*

- Develop an awareness of your own personal risk of burnout and secondary trauma and accept reactions as a normal response to specialised work [44, 51].
- Promote personal well-being by being physically active, engaging socially, and volunteering in your community and adopt appropriate self-care strategies [3, 48] (Box 1).
- Skill up in the field of trauma and family violence [47, 52].
- Take responsibility for your own mental health and well-being [49].
- Create opportunities to debrief with colleagues and decrease social isolation [48, 52].
- Develop and maintain clear client limits and boundaries [47, 48, 51].
- Recognise potential personality traits that may increase risk of experiencing secondary trauma [52].
- Find a sense of meaning in your work and develop a connection or spirituality to something that provides guidance for and develops a positive outlook on life [30].

* [my work is]….tremendously satisfying because I believe that my work is a little drop of water which when added to others over time will become a roaring mighty ocean that will spur the wind of change that will improve the lives of women everywhere (Respondent number: 37).*

## 5. Recognising Stress and Responding to It

Recognising that we are under stress can be difficult, particularly when professional cultures discourage it. Many PCPs have trained in systems that encourage them to be “in control,” rational rather than emotional and to cover feelings of helplessness and powerlessness [1]. This can be problematic when PCPs are stressed and hide it from their peers and colleagues and, conversely, can be an area that colleagues fail to see occurring. It has been suggested that partners, families, and friends are better at recognising when we are stressed than we are or our work colleagues are [1].  Table 1 the outlines common indicators of secondary stress.

### 5.1. Management of PCP Stress

While prevention is the best management, even with the best preparation some PCPs who work with family violence will become stressed. Management strategies can be drawn from both the trauma therapist and medical burnout literature.

Lee et al. related stress to the healthcare system, the occupational system, and the personal system [40]. The authors' previous work with researchers and staff who worked with sexual violence identified work-related management strategies and personal strategies as their main support framework [41].

In terms of the health care system, policies and procedures that recognise the time and expertise required for PCPs to support and provide long-term care for survivors are necessary. Ensuring that adequate counselling and effective therapy services are available to adults and children within the community is essential to support the work of the PCPs. In addition, ongoing PCP education must develop and reinforce basic knowledge skills and attitudes to ensure that they have the appropriate skills to recognise and support those who experience family violence and to enable these patients to access the appropriate trauma services [4].

 Work-related management strategies in managing traumatic material include ongoing development of skills because studies suggest that becoming experienced and an expert reduced trauma [5]. Spreading the workload where possible is reported as helping [41].

Those with better relationships with their coworkers and higher numbers of support systems were less likely to develop secondary traumatic stress [5].

Self-care strategies assisted PCPs; these included physical fitness, psychological strategies, spirituality/advocacy and staying connected. Various forms of physical activity and eating well have been described as well as using humour and laughter, avoiding retraumatizing material like films and newspapers, being pleased with small changes, being creative, travelling and exploring new places, gardening, talking with partners and family, eating and spending time with family and friends, going to church, praying, making a difference, and writing books, papers, and pamphlets to help [4, 52, 53].

Elwood and colleagues while recognising the importance of responding to workplace health risks and secondary trauma suggest that we should proceed with some caution [54]. The development and implementation of workplace policies on secondary and vicarious trauma must be informed by evidence and with cognisance of available resources. Much of the research done to date is limited to high-income settings and based on unclear definitions and weak methodologies. Elwood et al. recommend more methodologically sound; analytical research on secondary trauma, its symptoms, and responses to ensure that we do not waste limited resources on implementing ineffective interventions that are based on a poorly defined construct. Interventions must be culturally appropriate and implementable in resource-constrained environments.

## 6. Conclusion

Addressing the educational needs of PCPs responding to trauma is paramount. These need to be addressed at the undergraduate, prevocational, and practitioner levels on an ongoing basis to develop a skilled and competent workforce where the potential for stress is minimised.

As PCPs engage more with recognising and responding to family violence, it will be necessary to put in place the appropriate education and healthcare system, workplace, and personal support to enable the PCPs to continue to work with patients who have experienced family violence and their ongoing chronic health needs. PCPs need to be aware of their own support needs and the strategies they can put in place to engage well with those who have experienced family violence, providing safe long-term chronic care and access to the required services.

## Figures and Tables

**Box 1:**
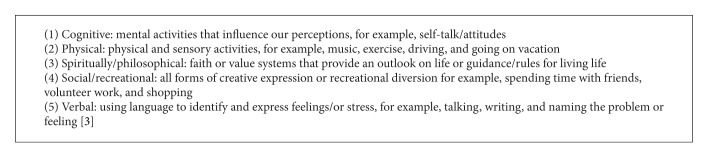
Five common self-care strategies identified in the literature.

**Table 1:** Indicators of secondary stress [2].

Psychological	Cognitive shifts/altered world view	Relationship changes
Continuing distressing emotions, for example, sadness, anger, fear, and shame	Loss of trust and sense of safety	Overidentification with patients
Intrusive images of the patient's experience, for example, nightmares, or flashbacks	Heightened sense of vulnerability	Detachment from patients, particularly those with traumatic experiences
Numbing or avoidance, for example, Being unable to ask about or work with family violence	Helplessness	Isolation from colleagues and peers
Somatic disorders, for example, headaches and abdominal pain	Loss of personal control	Overprotection of or detachment from family and friends
Addictive behaviours, for example, overworking, substance abuse, and compulsive eating.	Loss of sense of freedom	
Chronic physiological hyperarousal	Increased dependence on others	
Impaired day-to-day functioning, for example, chronic lateness, cancelling of appointments, and feeling unappreciated	Loss of confidence and job satisfaction	
